# Advances in artificial intelligence to predict cancer immunotherapy efficacy

**DOI:** 10.3389/fimmu.2022.1076883

**Published:** 2023-01-04

**Authors:** Jindong Xie, Xiyuan Luo, Xinpei Deng, Yuhui Tang, Wenwen Tian, Hui Cheng, Junsheng Zhang, Yutian Zou, Zhixing Guo, Xiaoming Xie

**Affiliations:** ^1^ Sun Yat-sen University Cancer Center, State Key Laboratory of Oncology in South China, Collaborative Innovation Center for Cancer Medicine, Guangzhou, China; ^2^ School of Medicine, Sun Yat-sen University, Guangzhou, China

**Keywords:** artificial intelligence, immunotherapy, deep learning, multi-omics, genomics

## Abstract

Tumor immunotherapy, particularly the use of immune checkpoint inhibitors, has yielded impressive clinical benefits. Therefore, it is critical to accurately screen individuals for immunotherapy sensitivity and forecast its efficacy. With the application of artificial intelligence (AI) in the medical field in recent years, an increasing number of studies have indicated that the efficacy of immunotherapy can be better anticipated with the help of AI technology to reach precision medicine. This article focuses on the current prediction models based on information from histopathological slides, imaging-omics, genomics, and proteomics, and reviews their research progress and applications. Furthermore, we also discuss the existing challenges encountered by AI in the field of immunotherapy, as well as the future directions that need to be improved, to provide a point of reference for the early implementation of AI-assisted diagnosis and treatment systems in the future.

## 1 Introduction

Tumor immunotherapy is the process of controlling and eliminating tumors by restarting the tumor immune cycle and restoring the body’s natural anti-tumor immune response. Immune checkpoint inhibitors (ICIs), chimeric antigen receptor T-cell therapy, tumor vaccines, and peripatetic immunotherapy are the main immunotherapy modalities currently used (1). These therapies, especially the use of ICIs such as PD-1 and CTLA-4, have achieved success in a major fraction of the patients, greatly enriching the prevailing clinical oncology treatments (2). However, it is still found in the clinic that some of the population is not sensitive to these drugs, and even the treatment outcome is not as good as traditional chemotherapy drugs. Therefore, it is crucial to screen the patients that will benefit from immunotherapy. While several of the current predictors such as PD-L1, tumor mutational burden, microsatellite instability, etc., do not sufficiently address this issue (3, 4).

In recent years, the application of artificial intelligence (AI) in the medical area has expanded significantly (5). Examples include surgical robots, which have proven to offer distinctive advantages. Scholars have made numerous attempts to apply AI to predict the efficacy of immunotherapy, for instance, by establishing immunotherapy prediction scores to predict treatment efficacy and effectively screen patients who can benefit from immunotherapy (6).

## 2 The overview of artificial intelligence to predict immunotherapy efficacy

**Figure 1:**
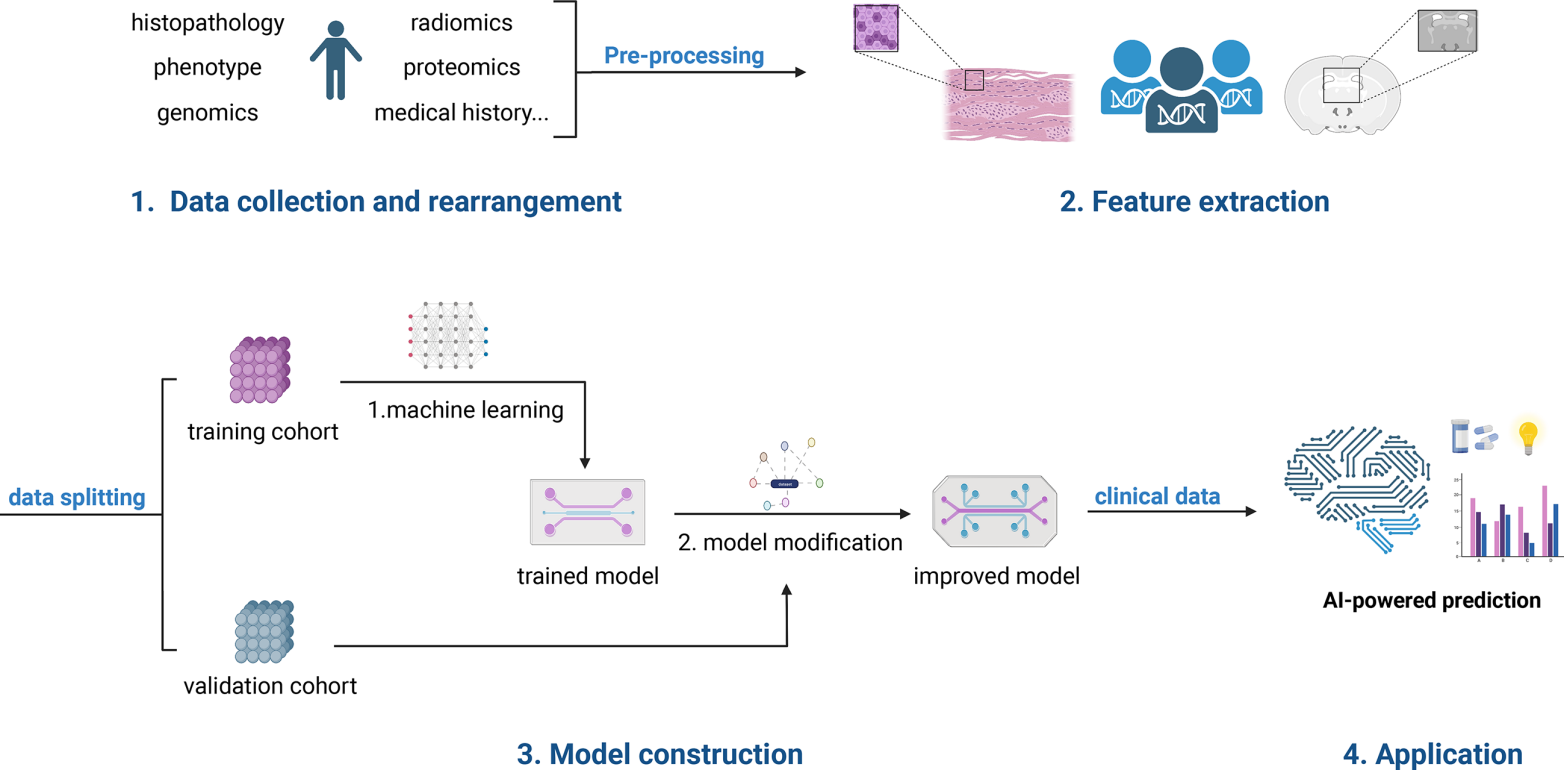
The workflow for using AI to predict immunotherapy efficacy. The First step is to collect multiscale medical data which include pathological tissue, CT/MR imaging-omics, genomics, proteomics, and more. The following steps are to gather, filter, segment, extract and select features. Then split these data into a training cohort and a validation cohort. Next, take the data from the training cohort, handing them over to the AI for learning and modeling. And then utilize the validation cohort to verify the learning results. Finally, a clinically applicable model will be developed.

The general strategy for using AI to predict the efficacy of immunotherapy **(** **Figure 1** **)** is to set up a training cohort and a validation cohort, take the multi-scale medical data from the training cohort, acquire, filter, segment, extract and select features, hand them over to the AI for learning and modeling, and then utilize the validation cohort to verify the learning results (7, 8). This multiscale medical data may include pathological tissue, CT/MR imaging-omics, genomics, proteomics, and more. The desired learning outcome is for the AI to be able to forecast whether a patient will benefit from immunotherapy or, at the very least, recommend which patients require more evaluation, like WGS. It’s also used to predict which immunotherapy drug will be most effective for the patient.

## 3 The existing approaches to predicting immunotherapy outcomes

### 3.1 AI predicts immunotherapy efficacy by histopathological features

The gold standard for tumor identification is histopathological tissue sections, which also provide a wealth of information that can be utilized to determine disease progression, select individualized treatment plans, and predict patient survivorship. However, due to the enormous labor required of experts to extract information from complex images, traditional histopathology procedures are unable to satisfy the demands of precision medicine (9). Currently, AI-based digital pathology has been successfully used in tumor diagnosis and treatment. This technology has a wide range of future applications, including improving the accuracy of pathological diagnosis, formulating treatment plans, predicting patient prognosis, and reducing manual workload, and more (10). For instance, AI can segment and identify tumor cells in pathology slides and accurately quantify immunohistochemical staining results (11). Thus, machine learning techniques based on histopathological analysis provide novel strategies to predict response to tumor immunotherapy (12). Among these, immunohistochemical (IHC) analysis, tumor-infiltrating lymphocyte (TIL), tumor-stroma ratio (TSR), and microsatellite instability are extensively researched.

Tumor cells expressing PD-L1 can suppress the immune response by binding to PD-1 on T cells, and ICIs is an anti-tumor strategy to block this interaction. Studies have shown that PD-L1 expression levels correlate with immunotherapy response and clinical results. Previous research has demonstrated that an AI-driven PD-L1 tumor ratio score-based analyzer can identify non-small cell lung cancer (NSCLC) more effectively than a pathologist can when predicting the immunotherapy response, and its results are objective and repeatable without human error (13). In addition, the absence of DNA defective mis-match repair (MMR) mechanism caused by mutations in the MMR gene results in the accumulation of somatic genomic mutations, which are closely associated with the ICB response (14). For MMR-deficient and MMR-proficient colorectal tumors, Le et al. (15) discovered immune-related objective response rates of 40% and 0%, respectively, suggesting that MMR status can be used to predict clinical response in patients treated with immune checkpoint inhibitors. In further, many studies have confirmed that higher levels of T-cell infiltration and T-cell counts are related to improved immune checkpoint blockage (16).

Microsatellite refers to several short, repetitive DNA sequences in the genome. Microsatellite instability is associated with DNA mismatch repair and involved in the development of many malignancies. Tumors with high microsatellite instability were found to respond well to immunotherapy (17). Kather et al. (18) demonstrated that deep learning has the potential to rapidly and accurately screen patients suitable for immunotherapy by precisely identifying the microsatellite stability status of patients with gastrointestinal cancer. Furthermore, AI can recognize lymphocytes, tumor cells, and mesenchymal stroma in the section and use three-dimensional reconstruction to highlight the spatial distribution of different cell types, which is another potential factor to evaluate the efficacy of immunotherapy (19).

TIL is closely associated with immunotherapy, and deep convolutional neural network models can be used to determine its distribution from eosin-hematoxylin (HE) stained images. By assessing the immune cell types in the tumor tissue matrix and classifying patients into types A and B, Zheng et al. (20) constructed a prognostic-relevant immune phenotype classifier. Higher levels of ICB were observed in phenotype A, which indicated a superior immunotherapy effect and prognosis. Additionally, there is an end-to-end strategy to train DL directly on response or outcome data, which used convolutional neural networks (CNNs) or graph neural networks (GNNs) to predict immunotherapy responses. According to these trials, the AUCs for predicting responders in lung cancer and melanoma were 0.778 and 0.69, respectively (21).

Tumor mutational burden (TMB) is defined as the number of non-synonymous single nucleotide variants (NsSNV) on tumor cells that can be transcribed into new antigenic peptides and presented on the cell surface, thereby activating T cells (22). High TMB is shown to be a predictive biomarker for lung cancer (23). Recent research suggests that deep learning systems can be used to predict immune responses. The study used HE-stained images to predict the status of TMB and reached an AUC of 0.78-0.98 in the external validation group, much higher than utilizing clinical data alone (24).

**Figure 2:**
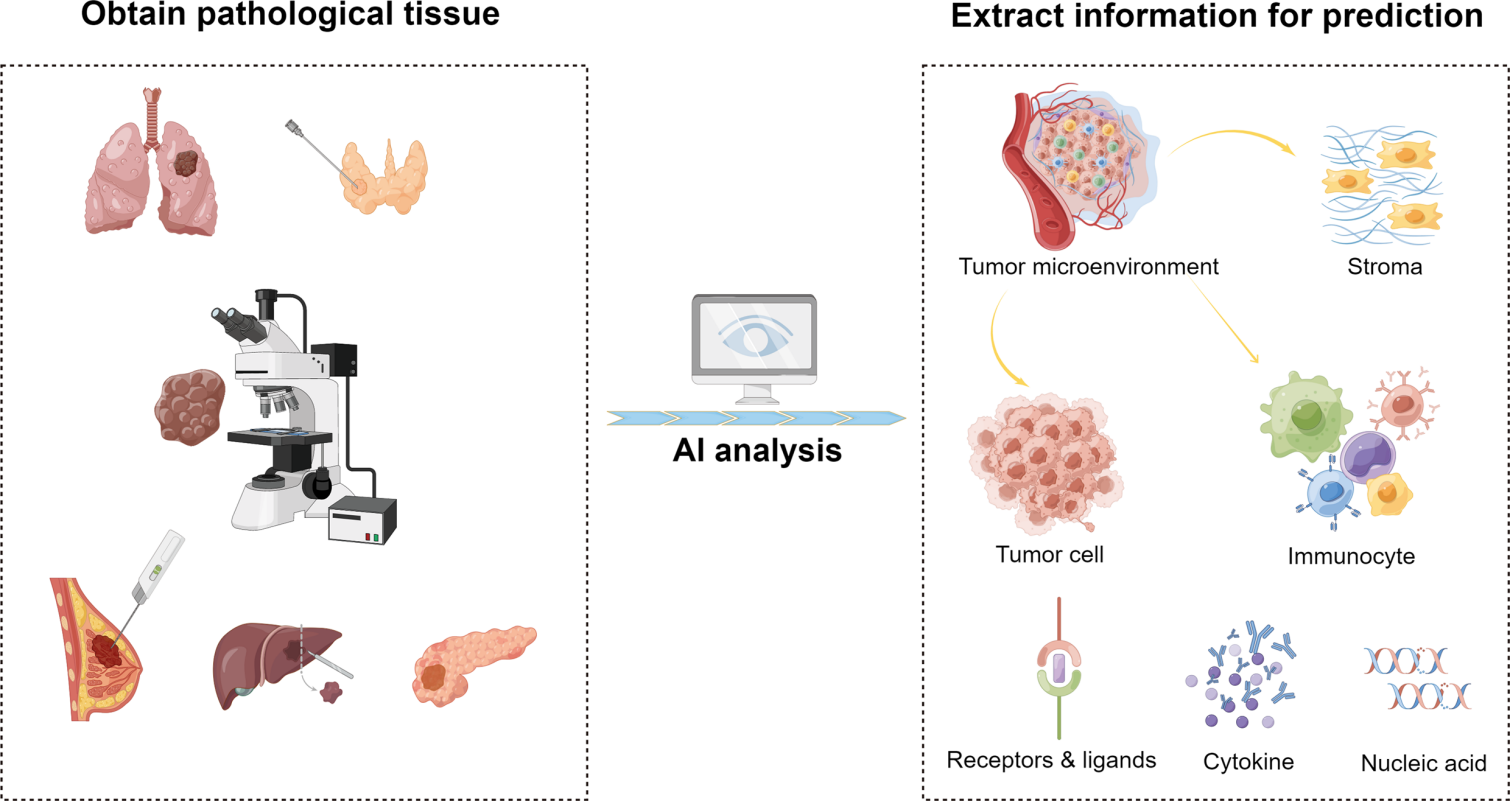
AI-based evaluation of immunotherapy efficacy by histopathological features. This is an illustration of the use of AI to forecast the effectiveness of immunotherapy. With the help of AI, more specific information can be extracted from clinical pathological tissues, including components of the tumor microenvironment and small molecular components like receptors, ligands, cytokines, nucleic acids, etc. From these elements, AI gathers data related to immunotherapy to forecast the efficacy.

AI has a promising development in predicting tumor immunotherapy response and can examine the state of tumor development through pathological data. Its algorithm and analysis results can be highly standardized and shared, which may assist advance medicine and actualizing precision medicine **(** **Figure 2** **).**

### 3.2 AI predicts immunotherapy efficacy by imaging-omics features

With the constant advancement of medical imaging equipment and technology, medical images are no longer simply pictures or restricted to the traditional “computer assisted diagnostics (CAD)”, but also contain high-throughput mineable data that are not recognizable to the naked eye (25). Imaging histology refers to AI-based imaging characterization that can provide more detailed information than single graphics can, displaying macroscopic, molecular, and cellular features (26, 27). Additionally, AI-based predictive models can provide reliable non-invasive biomarkers for evaluating immunotherapeutic response. Among various biomarkers, PD-L1 expression has been well validated in immunotherapy, and a combined model based on CT radiomics and clinical features can assess PD-L1 expression levels non-invasively (28). Sun et al. (29) selected 135 patients with tumors at different sites derived from phase I PD-1/PD-L1 monotherapy clinical trials. They established a predictive imaging model based on CT imaging by combining enhanced CT images with RNA-seq genomic data from tumor biopsy tissues. This model can analyze tumor-infiltrating CD8 signals and distinguish between immune-infiltrating and immunodetect types, making it an efficient method for predicting clinical outcomes of patients with advanced solid tumors after immunotherapy. Mu et al. (30) analyzed baseline PET/CT data of 194 patients with stage IIIB-IV NSCLC treated with PD-1/PD-L1 inhibitors, and they developed multiparametric imaging histological signature models which successfully predict whether patients would receive sustained clinical benefit from immunotherapy. However, further research is still required to determine whether models utilizing several data sources can perform better than models that simply use radiomics.

TMB is also well known for being one of the important indicators of ICI efficacy. He et al. (31) used deep learning techniques to analyze CT images from patients with advanced NSCLC and establish TMB radiomic biomarkers, which have high predictive value for ICI treatment response, overall survival (OS), and progression-free survival (PFS). Furthermore, Trebeschi et al. (32) used AI techniques to study the pre-treatment enhanced CT image analysis of patients with progressive malignant melanoma and NSCLC treated with PD-1, and they discovered that lesions with more heterogeneous morphology, which means compact borders and inhomogeneous density, were more likely to respond to immunotherapy. Additionally, there is a substantial association between imaging histological indices and mitosis-related pathways, suggesting that higher proliferative potential may signal better immunotherapy efficacy.

However, despite the fact that hyperprogression—rapid tumor progression following immunotherapy—is associated with a poor prognosis, there are no validated biomarkers to identify patients at risk for it (33, 34). Vaidya et al. (35) retrospectively summarized clinical and imaging data from 109 patients with advanced NSCLC treated with PD-1/PD-L1 immunosuppressant monotherapy, 19 of whom showed hyper progression. And researchers extracted textural features from the patients’ baseline CT images reflecting the texture within and around the target lesion as well as histological features quantifying the degree of peri-lesion vascular tortuosity, which can somewhat predict whether patients will develop hyper progression.

In general, the majority of studies’ Radiomics Quality Scores (RQS) ranged from 11 to 20 out of a possible maximum score of 36 points (36). It indicates that AI-based imaging-omics analysis can delve into the spatiotemporal heterogeneity of tumors and plays an important role in predicting immunotherapy response, biomarker expression, and patient prognosis, particularly in the absence of histopathological specimens. Deeper studies of imaging histology can aid in the diagnosis of immunotherapy-eligible patients, disease risk assessment, and precision medicine.

### 3.3 AI predicts immunotherapy efficacy by genomics

Thanks to advances in sequencing technology, a large amount of cancer genomic data has now been accumulated, providing more precise recommendations for directing tumor immunotherapy. The development of next-generation sequencing (NGS) technologies has enabled comprehensive genomic and transcriptomic screening. This allows to the generation of datasets for analyzing tumor drivers, as well as sequencing cancer cells, stromal cells, and immune cells within the tumor microenvironment to reveal the characteristics of therapeutic effects (37). The human genome contains more than 3 billion base pairs, making it a vast space of high-dimensional data with very complicated information. Whole genome sequencing (WGS) offers the most comprehensive information about the genome, but sorting out the genes, phenotypes, and their inter-regulatory relationships requires the assistance of AI, particularly deep learning techniques. Xie et al. (38) developed a predictive model that integrated genomic data from multiple perspectives, such as TMB, microsatellite instability, and somatic cell copy number variation in many different tumor types to effectively differentiate between “cold” and “hot” immune patients. The model was further externally validated using data from clinical trials, showing that patients in the hyperimmune group were more responsive to immunotherapy and had a better prognosis. By sequencing WES and RNA sequences from 110 patients with metastatic melanoma, Van Allen et al. (39) found that individual response rates to anti-CTLA4 correlated with TMB and cytolytic markers. It has also been shown that the expression and variant levels of platelet-related genes are closely related to the prognosis and immunotherapy response in patients with triple-negative breast cancer (40). These studies provided a direction to explore the variable response to immune checkpoint inhibitors and the identification of prognostic biomarkers.

Moreover, some factors in the transcriptome are still required to be added to the genomic analysis in order to fully predict immune response and explain drug resistance (41). By combining exome and transcriptome sequencing with mass spectrometry, Yadav et al. (42) discovered immunogenic mutant peptides with MHC specificity that could be useful for individualized vaccine development. Furthermore, Mo et al. (43) developed a high-throughput screening platform using 384-well plates to observe tumor immune interactions by co-culturing peripheral blood mononuclear cells (PBMC) and cancer cells in each well to assess cellular value-added and viability and to detect cell growth phenotypes. They also tested the effects of multiple bioactive compounds and identified three potential antagonists for enhancing immune activity. Some scholars have also proved it by epigenetic profiling that the DNA methylation landscape of patient CART19 cells influences the efficacy of the cellular immunotherapy treatment in patients with B-cell malignancy (44).

### 3.4 Others

Numerous studies have utilized AI for a variety of tumor immunotherapy applications (**Table 1**). The current liquid biopsy technology offers a more accessible and flexible approach to tumor diagnosis and treatment, which is represented by the detection of circulating tumor cell DNA. In immunotherapy, liquid genetic biomarkers are increasingly being developed to predict the therapeutic efficacy of ICIs (59), and molecular biological information in liquid specimens can be automatically identified and detected by AI technology (45). Additionally, some biomarkers are used to exclude hyperprogressive or pseudoprogressive disease after immunotherapy, including plasma cytokines interleukins and circulating tumor cell DNA (60).

**Table 1:** Various strategies that have been shown to predict immunotherapy outcomes with AI.

Prediction method	Forecast indicators	Outcomes	References
Liquid biopsy	Circulating tumor cell DNA, cytokines, serum complement levels, etc.	Circulating tumor cells can be used as a real-time detection system for targets such as PD-1; falling circulating tumor DNA was positively correlated with improved overall survival; serum C1q and LDH levels were correlated with the efficacy of immunotherapy	(45–47)
Multi-omics data	Genomics, transcriptomics, epigenomics, proteomics, radiomics, etc.	Multi-omics-based AI models present a chance to comprehend the information flow behind the disease. It can assess if each component of the model promotes the disease individually or whether they work together to treat it.	(48–50)
Clinical data	Population baseline data, medical history, examination results, etc.	Predictive models that distinguish immunotherapy age responders from non-responders can be constructed using data on patient age, sex, medical history, conventional laboratory tests, and follow-up CT scans	(51)
Tumor organoids	Tumor microenvironment, immune-tumor interaction, etc.	Organoids are very similar to the original tumor tissue, which can better mimic the *in vivo* immunotherapy response and observe the efficacy	(52, 53)
Others	miRNA abnormalities, gene mutations or recombination, gut microbes, etc.	The miRNA expression levels, ALK rearrangement, EGFR mutation, and gut microbial diversity were all related to the effect of anti-PD-1 treatment	(54–58)

AI, artificial intelligence; PD-1, programmed cell death protein 1; DNA, deoxyribonucleic acid; C1q, complement component 1, q subcomponent; LDH, lactate dehydrogenase; CT, computed tomography; miRNA, micro ribonucleic acid; ALK, anaplastic lymphoma kinase; EGFR, epidermal growth factor receptor.

In addition to genomics and imaging, proteomics technologies have been extensively explored to identify biomarkers for the effectiveness of tumor immunotherapy (46). An AI-based serum proteomics test model has been developed to predict response to ICIs in patients with metastatic melanoma (48). The application of multi-omics-based AI models to predict tumor immunotherapy responses is also promising. Multi-omics integrates genomics, transcriptomics, epigenomics, proteomics, and radiomics, allowing for a more thoroughly characterization of the disease with data from different sources (49). Based on the multi-omics concept, Yi Yang et al. (50) constructed a deep learning-based 90-day prediction model using age, gender, medical history, baseline data, routine laboratory tests, and follow-up CT scans of NSCLC patients to better distinguish immunotherapy responders from non-responders. Similarly, Arsela Prelaj et al. (61) used machine learning techniques to predict OS and PFS in responders and non-responders in a real-world study. Additionally, Peng Song et al. (51) suggested combining DNA and RNA sequencing, immunohistochemical staining results, demographic baseline data, medical history, and laboratory test results to analyze the information related to efficacy and break the limitation of relying only on PD-1 expression level and TMB level to predict immunotherapy response, and develop a mature model for immunotherapy response prediction for Chinese lung cancer patients. Along with PD-L1 expression, TMB, and TIL, it has been demonstrated that miRNA abnormalities (62), EGFR mutations (54), TP53 mutations (63), ALK rearrangements (55), gut microbiota (56), Fc gamma receptor (FcγR) polymorphisms (57) and serum complement levels (58) all influence immunotherapy response to some extent. It would facilitate the robustness of prediction if this information could be merged with pathological sections and imaging histology to form multi-omics integrated data.

Moreover, tumor-like organs serve as a useful screening model for immunotherapy because they can accurately reproduce the tumor microenvironment while still generating *in vitro* immune-tumor interactions (47). Incorporating AI into organoids is anticipated to overcome the safety and individualization challenges of conventional prediction by establishing a productive platform for tissue collection, tumor *in vitro* culture, growth analysis, and medication screening (52). AI could also forecast ICB responses by focusing on antigen presentation pathways (64) and cell necroptosis index (CNI) (53).

## 4 Future directions

**Figure 3:**
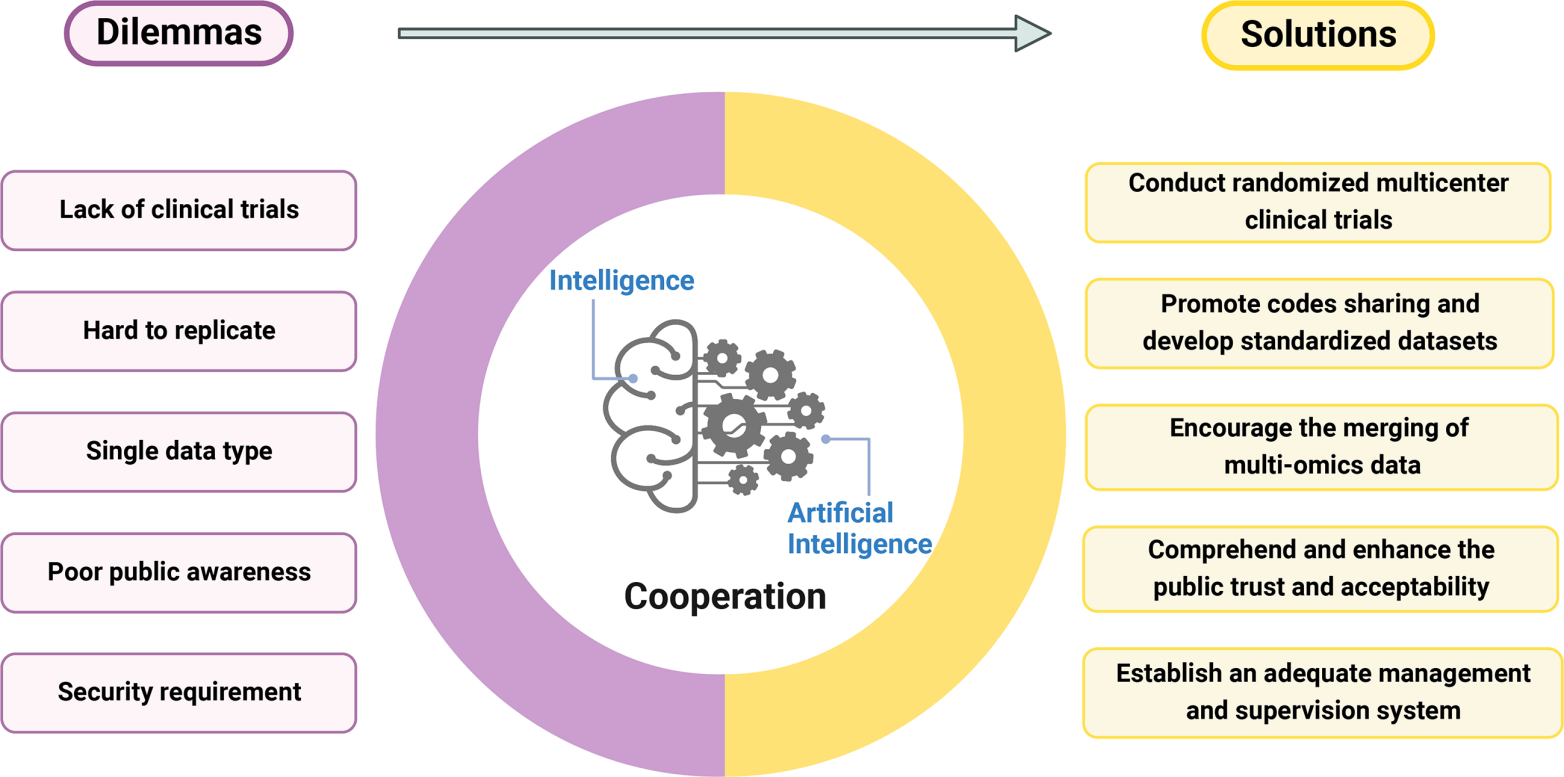
The dilemmas and solutions in predicting immunotherapy efficacy with AI. The left side outline the current difficulties AI encountered, and the right side proposes possible solutions. And only the excellent cooperation of artificial intelligence and human intelligence can achieve the best prediction effect.

Although the majority of studies have proven that its models have performed as well as or better than doctors, there are only a few successful real-world applications (65). Lack of a uniform database, industry standards, specialized clinical application situations, policy and regulatory assistance, and so on may all contribute to implementation challenges (**Figure 3**).

Firstly, there are relatively few prospective research and randomized clinical trials pertaining to the use of AI in immunotherapy, and these studies are highly biased. Therefore, we should increase experimental design transparency, strengthen the correlation with clinical reality, and reduce systematic error. Additionally, we must protect patient interests, minimize research waste, consider the cohort’s rationality, and avoid irrationally exaggerating research findings and potential applications.

Second, due to the scarcity of codes and data sources, AI models are challenging to fully replicate. Additionally, it is challenging to evaluate the stability of different models because of various inconsistent preprocessing methods and prediction objectives. Moreover, biomarkers may differ from primary tumor to metastatic tumor (66), making determining the optimal prediction for a certain tumor type much more difficult. Therefore, we should promote code sharing across fields and the development of standardized datasets.

Thirdly, the merging of multi-omics data must be encouraged to create a medical system that is more precise, individualized, and predictable. AI has a unique recognition model that can quickly identify and combine vast amounts of information in a way that humans cannot. An ideal AI-based predictive model for immunotherapy should include all relevant clinical information about the patient (genomics, imaging, proteomics, pathological tissue, demographic information, medical history, etc.). Since concepts like pan-cancer analysis have been reflected in the evaluation of PD-1/PD-L1 efficacy (67), it is important to promote the integrity and objectivity of data collection to facilitate the sharing of large data from multiple centers. This shows great promise for the future of immunotherapy (68).

Fourth, we need to comprehend how patients, medical professionals, and the general public feel about the use of AI in the healthcare system. To encourage the application of AI in practical uses, we should also carefully consider the fee-for-service model and deal with the common interests of patients, health insurance, doctors, and information engineers. Additionally, a strong management and supervision system must be established to reduce actual hazards and guarantee the security of this developing system (69, 70).

Last but not least, a reasonable legal and ethical environment should be established to facilitate the sharing of big data and the multi-regional and multi-centered use of AI systems, ensuring that they are ethical and that the corresponding responsibilities of each sector are clear.

In summary, due to the complexity of immunotherapy prediction, scientific researchers, enterprises, and clinicians must collaborate to build databases and industry standards, remove technical barriers, and support the development of AI-assisted systems that can precisely identify the target population of immunotherapy, accurately predict the efficacy and prognosis, and promote the implementation of AI-assisted treatment while winning the trust of both doctors and patients.

## 5 Conclusion

With the aid of artificial intelligence, a cutting-edge technology, it is now possible to treat tumor patients on an individual basis by automating the prediction of tumor immunotherapy effects based on constructed models. However, it still faces many challenges and dilemmas. Future AI-assisted systems are anticipated to be better able to model tumor biological behavior and medication treatment response, which would ultimately help the vast majority of tumor patients and enhance medical effectiveness and quality.

## Author contributions

XX, ZG, and YZ provided direction and guidance throughout the preparation of this manuscript. JX, XL, and XD wrote and edited the manuscript. JX, XL, XD, YT, and WT reviewed and made significant revisions to the manuscript. HC, and JZ collected and prepared the related papers. All authors contributed to the article and approved the submitted version.
